# SPION-mediated soil DNA extraction and comparative analysis with conventional and commercial kit-based protocol

**DOI:** 10.1007/s13205-014-0232-y

**Published:** 2014-06-24

**Authors:** Tanima Paul, Semanti Basu, Keka Sarkar

**Affiliations:** Department of Microbiology, University of Kalyani, Nadia, West Bengal India

**Keywords:** Superparamagnetic iron oxide nanoparticle (SPION), Soil DNA extraction, 16S rDNA, Real-time PCR, Density gradient gel electrophoresis (DGGE)

## Abstract

Direct isolation of soil DNA comes as an emerging technology to understand the microbial diversity of a particular environment circumventing the dependency on culturable methods. Soil DNA isolation is tough due to the presence of various organic components present in soil which interfere in extraction procedure. Here, we report a novel direct soil DNA extraction protocol utilizing bare superparamagnetic iron oxide nanoparticles and its comparison with conventional and commercial kit-based soil DNA extraction methods. The quality, quantity and feasibility of the recovered DNA from all the three methods towards various molecular techniques were checked. Our magnetic nanoparticle-based soil DNA extraction successfully yields pure DNA without any RNA or protein contamination as revealed by the nanodrop spectrophotometer and agarose gel electrophoretic study. Different methods of soil DNA extraction were evaluated on the basis of PCR, denaturing gradient gel electrophoresis and real-time PCR. Soil DNA extracted using conventional method fails to carry out critical molecular biology techniques where as magnetic nanoparticle-based soil DNA extraction gave good results which is comparable to commercial kit. This comparative study suggests that protocol described in this report is novel, less time consuming, cost effective with fewer handling steps and yields high quantity, good quality DNA from soil.

## Introduction

Soil is an immense reservoir of microbial diversity and it is estimated to have 10^9^ cells per gram of soil (Whitman et al. 1998). Since most of the soil microbes are non-cultivable, the analysis of whole genome microbial diversity is restricted. To overcome the limitation of culture-dependent method emphasis is being made to encourage the development of culture-independent approaches (Head et al. 1998; Muyzer et al. 1993) to provide an overview of species richness in soil. This information of microbial diversity can be utilized for the study of community physiology, novel approaches in bioremediation and recycling, and discovering new biotechnology applications. The methods of direct DNA extraction from soil made dramatic improvements in analysis of soil microbial communities. But soil DNA extraction is difficult due to the presence of humic acids which are coextracted during DNA isolation and leads to inhibition of *Taq* DNA polymerase during PCR (Smalla et al. 1993), interfere with enzymatic restriction digestion (Porteous and Armstrong 1991), reduce transformation efficiency (Tebbe and Vahjen 1993) and DNA hybridization specificity (Steffan et al. 1988).

Extraction of DNA directly from soil includes two parts: (1) direct lysis of cells either by physical or chemical method or by enzymatic method or in combination among these three, and (2) separation of the DNA from suspension mixture containing lysed cell and soil particles. Various physical methods used for cell lysis are freeze-thawing or freeze-boiling (Degrange and Bardin 1995), bead beating (Bürgmann et al. 2001), mortar mill grinding (Tebbe and Vahjen 1993), grinding under liquid nitrogen (Volossiouk et al. 1995), ultra sonication (Picard et al. 1992), and thermal shock (Orsini and Romano-Spica 2001). Chemical method includes the use of detergents such as sodium dodecyl sulfate (SDS) in combination with heat treatment and EDTA or Chelex 100 as chelating agents (Herron and Wellington 1990; Jacobsen and Rasmussen 1992) or with diverse Tris buffer or sodium phosphate buffers (Krsek and Wellington 1999). Use of polyvinylpolypyrrolidone (PVPP) and cetyltrimethyl-ammonium bromide (CTAB) results in partial removal of humic substances (Krsek and Wellington 1999) but PVPP cause DNA loss (Zhou et al. 1996) and CTAB forms insoluble complexes with denatured proteins, polysaccharides and cell debris (Saano et al. 1995). Enzymatic lysis method includes the use of lysozyme (Tebbe and Vahjen 1993) and proteinase K to digest contaminating proteins (Zhou et al. 1996). Several methods for separating and purifying soil DNA from the mixture of soil and lysed cells are organic solvent extraction (phenol or chloroform) followed by ethanol, isopropanol or polyethylene glycol precipitation (Steffan and Atlas 1988) but are toxic. Another separating process is cesium chloride (CsCl) density gradient centrifugation but extensive purification results in DNA loss and did not remove all organic contaminants (Steffan and Atlas 1988). Another approach to eradicate organic contaminants during DNA extraction is the use of magnetic capture hybridization (MCH) (Jacobsen 1995), but MCH-based DNA extraction is restricted to specific DNA sequences only. Various types of resin columns (Amorim et al. 2008) and commercial kits are also present for soil DNA extraction but they are too expensive and not suitable for less sample volume or large sample number. All the processes described above have some common limitations such as time consumption, costly, multi-step and not efficient for eradicating inhibitors for PCR and other molecular techniques. Here we proposed a standard method which is cost effective, time saving and robust for extraction of ultrapure whole genomic DNA from soil approaching superparamagnetic iron oxide nanoparticle (SPION) and compared our process with conventional and commercial kit-based method of soil DNA extraction.

In particular, magnetic nanoparticles have gathered interest due to its large surface/volume ratio, biocompatibility and less toxicity (Ito et al. 2005). So far in our knowledge only a few attempts have been made to extract soil DNA using surface functionalized magnetic nanoparticle (Sebastianelli et al. 2008). Here, we have synthesized and characterized magnetic nanoparticle and used them to isolate soil DNA without any modification. Feasibility of isolated soil DNA towards PCR, DGGE and RT-PCR compatibility has been performed to investigate the quality of DNA which makes our method unique. Thus, we introduce a new method of soil DNA isolation which is suitable for molecular biology techniques.

## Methods

### Sample collection

Soil sample was collected from university campus at a depth of 5 cm from the ground using sterile spoon in sterile plastic bags. Sample was stored at 4 °C till further use. Debris of the soil was removed at the time of DNA extraction.

### Soil DNA extraction

#### Conventional method

Soil DNA was extracted from 0.5 g soil using phenol/chloroform method as described elsewhere (Zhou et al. 1996). DNA was finally suspended in 50 µl TE buffer and stored at −20 °C.

#### Commercial kit

Soil DNA was extracted from 0.1 g soil using commercial kit (SoilMaster™ DNA Extraction Kit, Epicentre) according to the manufacturer’s instructions. DNA was dissolved in 50 µl TE buffer and stored at −20 °C.

#### Using SPION

Superparamagnetic iron oxide nanoparticles (SPIONs) were prepared by chemical co-precipitation of Fe^2+^ and Fe^3+^ ions under alkaline conditions (Bandyopadhyay et al. 2011). Nanoparticles were dried in vacuum drier and dissolved in water for further use. Nanoparticles were characterized using Transmission electron microscopy (TEM; Tecnai S-Twin, FEI, Hillsboro, OR, USA), Superconducting Quantum Interference Device (SQUID; MPMS, Quantum Design Inc., San Diego, CA, USA) and Dynamic light scattering (DLS; Zetasizer, Malvern Instruments Ltd, Malvern, UK) to determine the size, magnetic property and zeta potential, respectively.

Soil DNA extraction was carried out by freshly prepared SPION sonicated (Hielscher Ultrasonics, UP50H) at 60 MHz for 20 min. Soil (0.5 g) was suspended in 1.5 ml lysis buffer (100 mmol/L Tris–HCl pH 8.0, 100 mmol/L EDTA pH 8.0 and 1 mmol/L NaCl) and 100 µL of 2 % W/V SDS added separately in a sterile 15 ml falcon tube. The sample was homogenized using vortex mixture to break the soil clumps and incubated at 65 °C for 30 min with end over end rotation. The mixture was centrifuged (Centrifuge: Z 36 HK—Hermle Labortechnik) at 3,000×*g* for 30 s with slow acceleration and deceleration. Supernatant was transferred to a fresh 2 mL microfuge containing 20 µL of magnetic nanoparticle to which binding buffer (20 % W/V polyethylene glycol—mol wt 6,000 and 4 mol/L NaCl) was added in equal volume to that of the supernatant. It was again incubated at room temperature for 5 min with end over end rotation. SPION were immobilized using magnet and supernatant was discarded. The particles were washed twice using 90 % and then 70 % ethanol and dried at room temperature. DNA was eluted in 50 µL TE buffer (10 mM Tris–Cl, 1 mM EDTA pH 8.3) incubating at 65 °C with continuous agitation and nanoparticles were captured by external magnetic field. Buffer containing the extracted DNA was transferred carefully into a fresh microfuge and stored at −20 °C.

### Comparison of isolated DNA in terms of quality and quantity

Agarose gel electrophoresis of isolated DNA from all the three processes was carried out in 0.8 % gel and observed under Gel Doc System (Bangalore Genei, Bangalore, India) to confirm the DNA extraction and length of the extracted DNA. Purity and yield of the extracted DNA were analyzed using Nanodrop 2000 spectrophotometer (Thermo Scientific), where *A*_260/280_ gives protein contamination and *A*_260/230_ gives other organic acid contamination mainly humic acid.

### PCR amplification

To verify the feasibility of the extracted DNA in molecular biology, PCR amplification of all the three soil DNA samples was carried out using 16S rDNA targeted primer pair 63f (5′-CAGGCCTAACACATGCAAGTC-3′) and 518r (5′-ATTACCGCGGCTGCTGG-3′) (Breugelmans et al. 2007). The PCR mixture contained 1 µL of DNA extract, 5 µL of 10X PCR buffer, 5 µL of 1 % bovine serum albumin (BSA), 4 µL of dNTPs (2.5 mM each), 0.25 µL of each primer (0.1 mM), and 0.25 µL Taq DNA polymerase (5 U µL^−1^). The final volume was made up to 50 μL using nuclease-free water. Amplification was carried out in 30 cycles in a thermal cycler (model 2700, Applied Biosystems, Foster City, CA, USA) as follows: 5 min; 94 °C, 30 s; 94 °C, 45 s; 56 °C, 1.30 min; 72 °C, followed by a final extension for 6 min at 72 °C. PCR products were analysed by 1 % agarose gel electrophoresis.

### Compatibility towards other molecular techniques

#### Denaturing gradient gel electrophoresis

To detect the microbial diversity in the soil sample, DGGE was done using the extracted soil DNA using all the three methods. For increased specificity, sensitivity and yield of PCR product, touchdown PCR was carried out prior to DGGE using 63f and 518r primer pair (forward primer with 40 bases GC clamping at 5′ end). The reaction mixture was same as mentioned above and the reaction condition was as follows: 5 min; 94 °C; 10 cycles of 94 °C for 30 s, 60–55 °C (0.5 °C decrease in each step) for 30 s, 72 °C for 30 s; 20 cycles of 94 °C for 20 s, 55 °C for 30 s and 72 °C for 30 s; final extension was at 72 °C for 7 min and then 4 °C storage. PCR products were checked in 1 % agarose gel electrophoresis.

DGGE was performed in a DCode System (Bio-Rad, Munich, Germany) with 8 % (w/v) acrylamide gel containing a denaturant gradient from 40 to 60 % of urea and formamide (100 % denaturant contains 7 M urea and 40 % (v/v) formamide). Running buffer used was 0.5X TAE buffer pH 7.8 containing 20 mM Tris, 10 mM acetate and 0.5 mM disodium EDTA electrophoresis was done at 60 °C for 8 h with 150 V. Staining was done with 0.5 µg/ml ethidium bromide solution and observed under Gel Doc System (Bangalore Genei, Bangalore, India).

#### Real-time PCR

Real-time PCR was carried out in MyiQ2-BioRad, using extracted soil DNA by magnetic nanoparticle and commercial kit to check its compatibility towards this molecular technique. Copy number of total bacterial 16S rDNA gene in the extracted soil DNA dilution was calculated from the equation obtained in standard curve preparation as mentioned elsewhere (Lee et al. 2008). Dilutions up to 10^−6^ of vector pTZ57R/T with a 1,465 bp insert of 16S rDNA was used as standard. Soil DNA samples were half diluted and used as template. Reactions of all the dilutions were performed in triplicate and mean value was considered. Total reaction mixture volume was 20 µL including 10 µL 2X master mix containing Syber Green (BioRad), 5 µL nuclease-free water, 0.5 µL of each primer (63f and 518r; 10 pmol) and 4 µL template DNA. Reaction condition was 5 min at 95 °C; 40 cycles of 20 s at 95 °C and 30 s at 60 °C.

## Results

### Characterization of the SPION

**Fig. 1:**
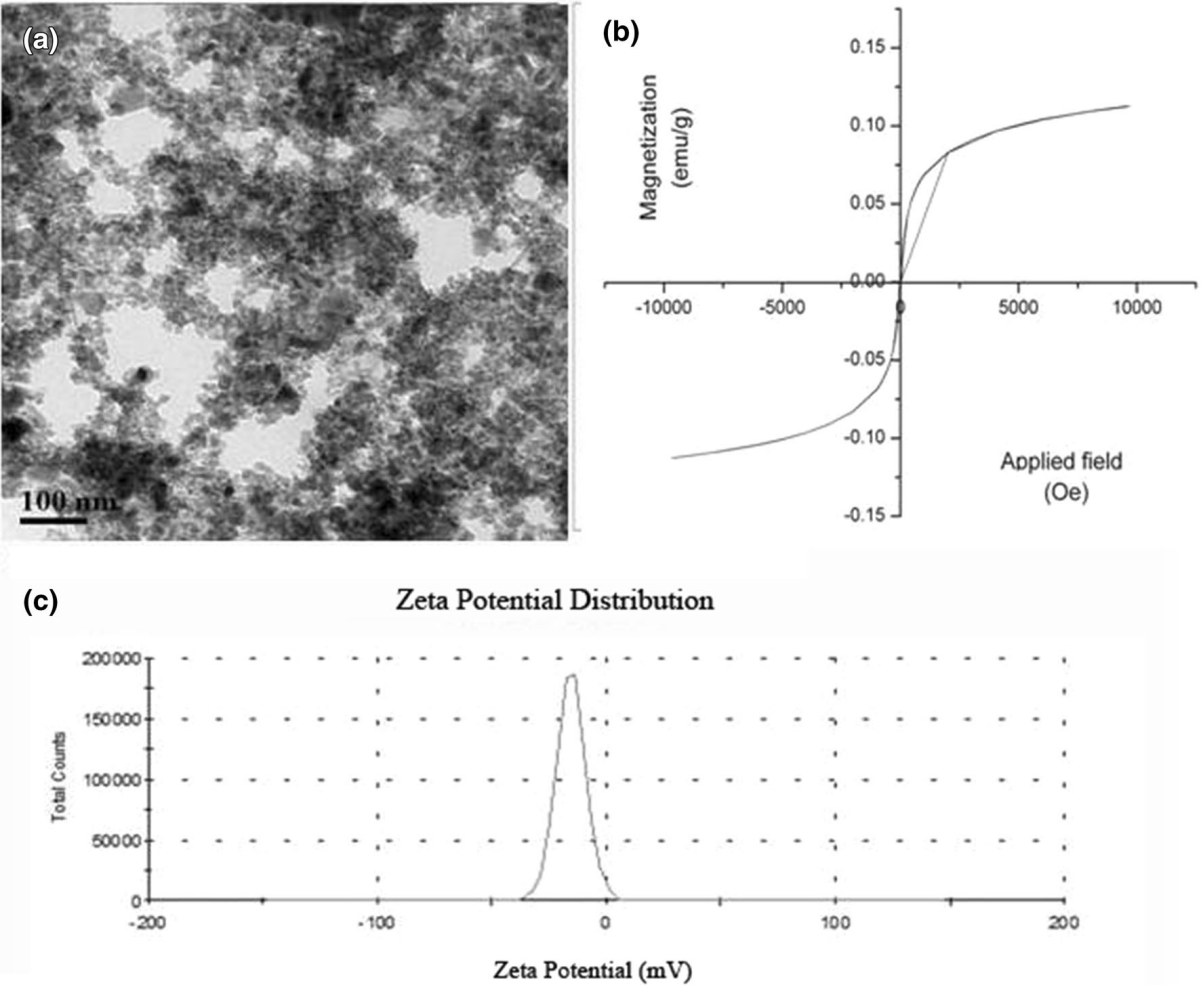
Characterization of superparamagnetic iron oxide nanoparticle (**a** TEM image; **b** SQUID data; **c** zeta potential)

Characterization of the prepared SPION made by transmission electron microscopy revealed the size of nanoparticle to be 8 nm (Fig. 1a). Absence of hysteresis loop in the *M*–*H* curve obtained from SQUID data confirmed the lack of magnetic remanence indicating superparamagnetic nature of the synthesized iron oxide nanoparticles (Fig. 1b). Zeta potential was found to be −15.04 mV (Fig. 1c). The large negative value suggested the small particle size as well as the stability of nanoparticles without agglomeration.

### Comparison of various soil DNA extraction process and quality analysis

Purity of nucleic acids is of great concern for carrying out the molecular techniques. Interference of organic compounds in soil DNA extraction is a great problem. Here, we have compared our method of soil DNA extraction using bare magnetic nanoparticle with the DNA extracted using conventional method (phenol/chloroform) and commercial kit. Both quality and quantity of the extracted DNA were checked using agarose gel electrophoresis and spectrophotometer, respectively. Values of *A*_260/280_ and *A*_260/230_ are listed in Table 1 for determining protein and organic contaminations, respectively. A high 260/230 ratio (>2) is indicative of pure DNA, while a low ratio is indicative of humic acid contamination. Similarly, a high 260/280 ratio (>1.8) is indicative of pure DNA, while a low ratio is indicative of protein contamination. From our experiment, *A*_260/230_ and *A*_260/280_ of extracted DNA using magnetic nanoparticle were found to be 2.01 and 1.76, respectively, indicating minimum protein and other organic contaminations.

**Table 1:** Comparison of various soil DNA extraction process

	Conventional method	Commercial kit	Using magnetic nanoparticles
Concentration (ng/µL)	2.5	8.9	9.89
Amount of DNA/g of soil (ng)	125	445	494.5
*A*_260/280_	1.54	1.78	1.76
A_260/230_	1.83	1.97	2.01
Time required	5 h	2 h 15 min	1 h 15 min
Cost in INR	250	436	20
Method	Difficult	Easy	Easy

**Fig. 2:**
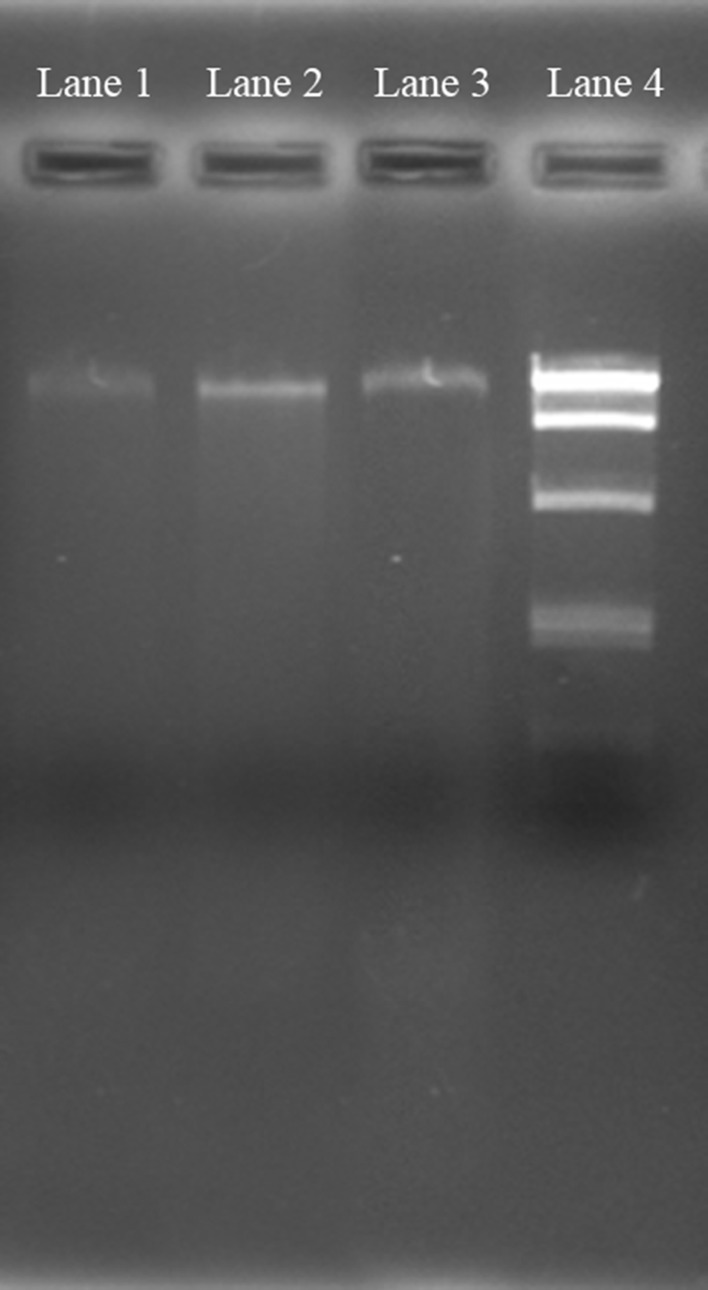
Agarose gel image of extracted soil DNA using various methods (*lane1* conventional method; *lane2* magnetic nanoparticle based; *lane3* commercial kit; *lane4* 1 kb)

Electrophoretic study of the extracted soil DNA from all the three methods is given in Fig. 2 (lane 1, conventional method; lane 2, magnetic nanoparticle-based method; lane 3, commercial kit; lane 4, DNA ladder). A major good intensity, discrete band was found around 10 kb in magnetic nanoparticle-based method whereas conventional method gave very faint band and commercial kit-based method gave moderate intensity band.

### Comparison of PCR amplification

**Fig. 3:**
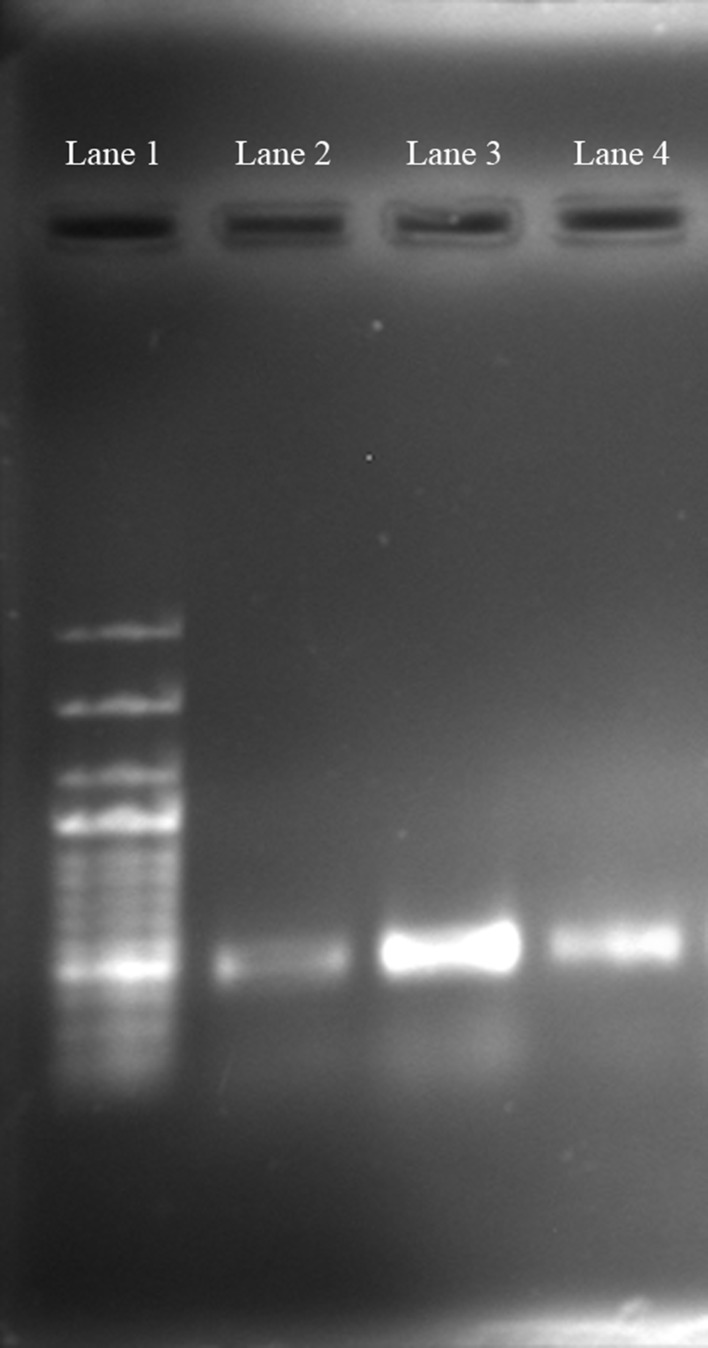
Agarose gel image of PCR amplicons of 16S rDNA gene using extracted soil DNA from various methods (*lane1* 100 bp DNA ladder; *lane2* conventional method; *lane3* magnetic nanoparticle based; *lane4* commercial kit)

Soil DNA extracted using all the three methods was checked for PCR compatibility, since, PCR amplification is the basic requirement to carry out any molecular biology technique. Agarose gel image of the PCR-amplified products from DNA extracted using all the three processes is shown in Fig. 3. A major intense band around ~450 bp was observed in magnetic nanoparticle-based soil DNA purification (lane 2) whereas conventional method restricts PCR amplification (lane 1). Considerable amplification was observed in commercial kit-based soil DNA extraction (lane 3).

### Compatibility towards other molecular techniques

**Fig. 4:**
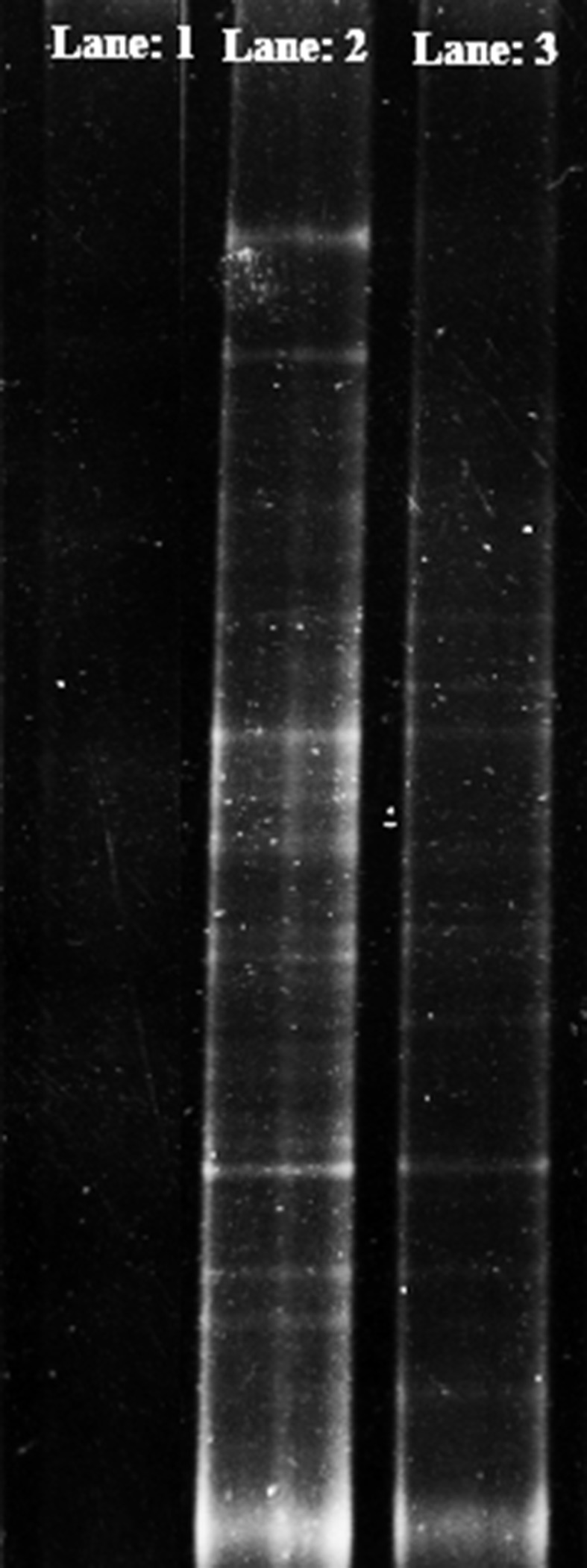
DGGE image of the amplified products showing a number of discrete bands indicating different members of soil bacterial community (*lane1* conventional method; *lane2* magnetic nanoparticle based; *lane3* commercial kit)

Denaturing gradient gel electrophoresis and real-time PCR are some of the important molecular techniques to overview bacterial diversity in particular environments. DGGE was performed using soil DNA extracted using all the three methods. DGGE profile (Fig. 4) showed apparent band differences in magnetic nanoparticle-based (lane 2) and commercial kit-based (lane 3) soil DNA isolation. Various major bands indicate the presence of predominant bacterial groups. Decreased number of bands in DGGE profile, Lane 3 indicates less extraction of initial soil DNA using commercial kit than magnetic nanoparticle-based isolation. Good band intensity in DGGE banding pattern reveals its applicability towards sequencing of the separate band. Conventional method fails to give any discrete band in lane 1. PCR product of both magnetic nanoparticle and commercial kit-based soil DNA isolation was satisfactory to carry out DGGE.

**Fig. 5:**
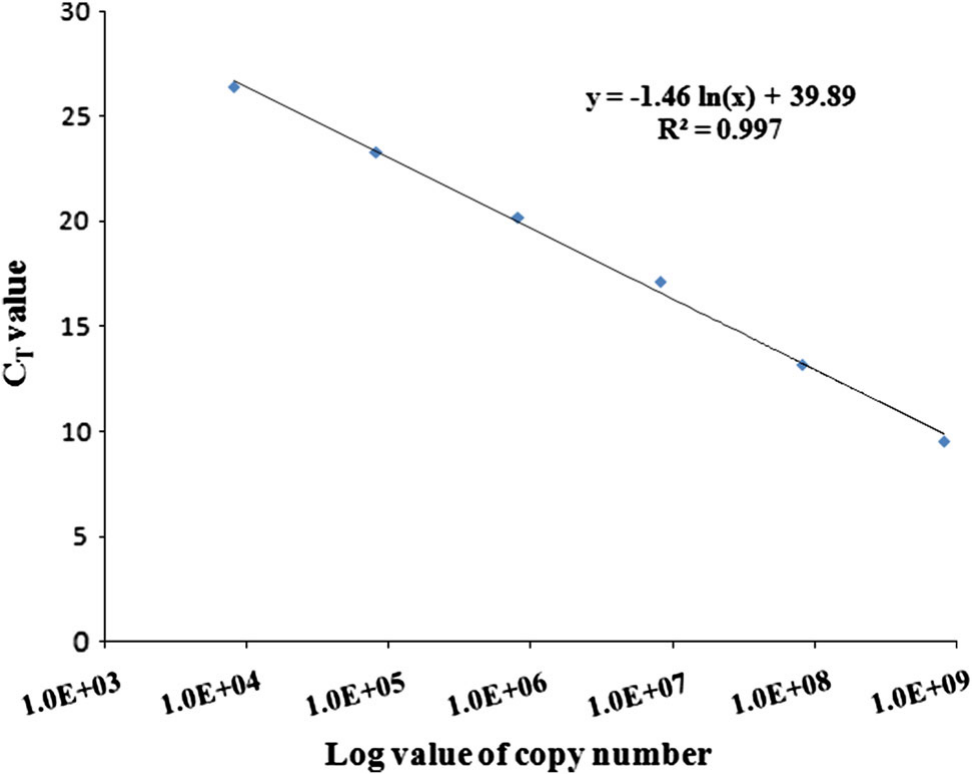
qPCR generated standard curve after plotting CT values and copy numbers of the standard samples

Only magnetic nanoparticle-based and kit-based soil DNA isolation was taken into consideration to carry out real-time PCR since conventional method fails to give PCR amplification required in this molecular technique. In real-time PCR all the dilutions of standards gave separate threshold (*C*_T_) values which were used for the calculation of copy numbers (Table 2) as described by Lee et al. (2008). Standard curve was obtained by plotting *C*_T_ values against logarithmic value of copy number (Fig. 5). *R*^2^ value of the slope gives amplification efficiency and it was found to be 0.99. Finally total copy number of bacterial 16S rDNA gene in extracted soil DNA using both magnetic nanoparticle and commercial kit was calculated by putting the *C*_T_ values 19.39 and 21.24, respectively, in the equation obtained from the standard curve. Total copy number of bacterial 16S rDNA gene in extracted soil DNA using both magnetic nanoparticle and commercial kit was 3 × 10^5^ and 8.75 × 10^4^ µL^−1^, respectively.

**Table 2:** *C*_T_ values and copy numbers of the standard samples

Samples	Std 1 (10^−1^)	Std 2 (10^−2^)	Std 3 (10^−3^)	Std 4 (10^−4^)	Std 5 (10^−5^)	Std (10^−6^)
*C*_T_ values	9.54	13.21	17.13	20.22	23.30	26.43
Copy numbers^a^	8.2E+08	8.2E+07	8.2E+06	8.2E+05	8.2E+04	8.2E+03

^a^ Copy number was detected using standard equation

## Discussion

**Fig. 6:**
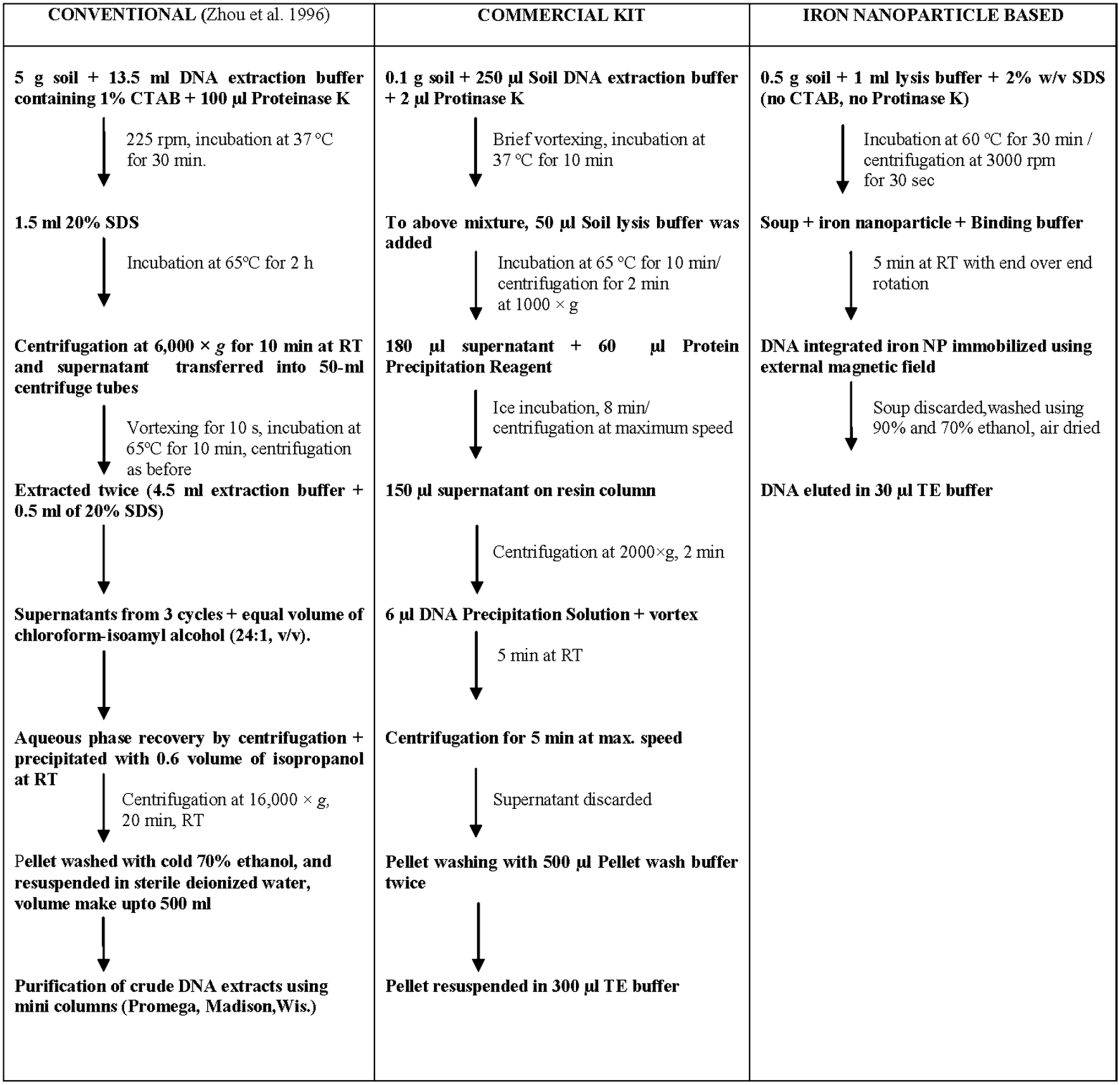
Comparison of magnetic nanoparticle-based soil DNA extraction with conventional and commercial kit-based method

Various methods used for soil DNA extraction can cause problems in molecular analysis of populations in natural environment due to variable efficiencies of different methods. DNA extraction from soil has two basic requirements: lysis of representative microorganisms and extraction of high molecular weight, inhibitor-free DNA for subsequent molecular techniques. It is important to select an extraction method which yields DNA of suitable quality and purity with cost effectiveness. In the present study, we have compared our novel magnetic nanoparticle-based soil DNA extraction process with conventional and commercial kit-based method. The main advantage of our method is cost effective and rapid. While conventional method and commercial kit require INR 250 and INR 436 per smaple, respectively, SPION-mediated soil DNA extraction serves this purpose only in INR 20 per sample. An overview of the whole study is given in Fig. 6. This indicates how multi-stepping, tedious and costly procedures of conventional and commercial kit-based method can be avoided by our iron nanoparticle-based soil DNA extraction method. Due to large surface/volume ratio and small size, nanoparticles are suitable for interaction with biomolecules (Ito et al. 2005). We have prepared magnetic nanoparticle of 8 nm size which are monodisperse. Absence of hysteresis loop in SQUID data indicates minimum magnetic remanence on removal of external magnetic field showing superparamagnetism. This superparamagnetic property is helpful for various biological applications such as separation of biomolecules (Bandyopadhyay et al. 2011).

Extraction of DNA from soil is tough due to the interference of various organic substances especially humic acid. Various method of soil DNA extraction are already present which include various enzyme treatments such as protinase K, lysozyme, freeze-thawing, bead beating, organic precipitation, etc. These methods are time consuming, costly, produce some chemical hindrance during amplification and amount of extracted soil DNA is very low. Here, we have compared the magnetic nanoparticle-based approach with the conventional and commercial kit-based soil DNA extraction processes on the basis of quality and quantity of DNA yield. Protein and organic component contaminations were checked at *A*_260/280_ and *A*_260/230_, respectively. Extracted DNA from various processes was also checked in agarose gel electrophoresis for DNA quality. Thus our investigation indicates that the conventional method yields minimum quantity of DNA whereas both kit-based method and our method yield good quality DNA. Kit-based soil DNA extraction is very much costly as compared to our method.

For conducting further molecular biology approaches, the quality of the extracted DNA from all the three methods was checked by amplification of 16S rDNA gene. In various cases, direct detection of microorganisms from natural environments was carried out using PCR for the study of microbial diversity (Bej et al. 1991; Wegmuller et al. 1993). It is of utmost importance to get an inhibitor-free DNA template for PCR amplification as co-extraction of organic pollutants especially humic acid hinder PCR amplification by damaging *Taq* polymerase. Our method successfully overcomes these limitations and gives a good amplification showing discrete band with no smear. We did not manage to amplify the 16S rDNA gene from DNA extracted using conventional method probably due to the presence of hazardous chemicals whereas commercial kit-based method gave good amplification.

Furthermore, DGGE and qPCR were performed to check the feasibility of the extracted soil DNA from all the three methods towards these molecular techniques. DGGE is a technique to study microbial diversity which provides an immediate display of population constituents in both qualitative and semiquantitative manner avoiding cloning. Since DGGE exploit PCR-amplified products, a good quality DNA template is required without any inhibitors. Touchdown PCR is generally preferred to avoid non-specific binding. PCR products of the soil DNA extracted using all the three methods were subjected to DGGE for analyzing the banding pattern. Conventional method fails to give any banding pattern due to poor PCR quality. Whereas, magnetic nanoparticle-based soil DNA extraction showed a good banding pattern with discrete bands. The commercial kit-based DNA extraction method also makes it possible to obtain DGGE profile, but the number of visualized bands and the signal fidelity are somewhat lower in this case.

Real-time PCR was also performed to quantify copy number of total bacterial 16S rDNA gene present in the soil sample using DNA extracted by our method and commercial kit. Total copy number of the bacterial 16S rDNA gene present in the extracted soil DNA was calculated by putting the *C*_T_ value in the equation developed from the standard curve. Compatibility of DNA template extracted by our method towards real-time PCR will help to determine microbial community structure in future. Thus our method of soil DNA extraction using magnetic nanoparticles proved to be a unique and successful method which can be extended to carry out various critical molecular biology techniques.

## Conclusions

In conclusion, we report a comparative study of existing conventional protocol and commercially available kit of soil DNA extraction with our method approaching magnetic nanoparticles. Our method yields a good quality, un-sheared, high amount DNA which is comparable to commercially available kit whereas conventional method yields poor quality DNA. Our method of soil DNA extraction needs no surface functionalization of the nanoparticle, no RNAase or protinase K treatment and does not require any organic solvent or hazardous chemicals. Magnetic nanoparticle-based soil DNA extraction does not involve any sophisticated instrument rather only a magnet can perform the extraction process under any laboratory circumstances. Soil DNA extracted by our method is potential in undergoing critical molecular biology techniques such as PCR, qPCR and DGGE which are utilized as major tools to explore microbial community of particular environments. Thus, we present a simple, less time consuming, cost effective method of direct soil DNA extraction which can contribute to study various microbial diversity and exploiting them for industrial, environmental and agriculture applications. Thus a repetitive experiment with diverse soil sample is required to extrapolate the work.
